# Quality of Life in Chronic Heart Failure With Obstructive Sleep Apnea: A Systematic Review

**DOI:** 10.1002/nop2.70385

**Published:** 2025-11-28

**Authors:** Francesco Limonti, Giovanni Cangelosi, Diaratou Dabre, Gianluca Marino, Arcangelo Correra, Sara Morales Palomares, Nicola Ramacciati, Francesco Gravante

**Affiliations:** ^1^ Department of Biomedicine and Prevention University of Rome Tor Vergata Rome Italy; ^2^ Experimental Medicine and ‘Stefania Scuri’ Public Health Department University of Camerino Camerino Italy; ^3^ Department of Translational Medicine University of Naples ‘L. Vanvitelli’ Napoli Italy; ^4^ Department of Strategic Services Local Health Authority of Caserta Caserta Italy; ^5^ Department of Pharmacy, Health and Nutritional Sciences (DFSSN) University of Calabria Rende Italy; ^6^ Department of Critical Care, San Giuseppe Moscati Local Health Authority of Caserta Aversa Italy

**Keywords:** chronic heart failure, obstructive sleep apnea, quality of life, sleep apnea, systematic review

## Abstract

**Aim:**

To describe the quality of life in patients with chronic heart failure and coexisting obstructive sleep apnoea, and to examine the effect of common sleep‐disordered breathing treatments on quality of life, functional capacity and cardiac outcomes.

**Design:**

Systematic review.

**Methods:**

This systematic review followed the Preferred Reporting Items for Systematic Reviews and Meta‐Analyses guidelines and was registered in PROSPERO (CRD42025634352).

**Data Sources:**

A comprehensive search was conducted in PubMed, CINAHL, Scopus and Web of Science up to March 2024. Study quality was assessed using the Joanna Briggs Institute Critical Appraisal Checklists.

**Results:**

Fourteen studies were included, involving 2048 patients with chronic heart failure and sleep‐disordered breathing, including obstructive and central sleep apnea. Sleep‐disordered breathing was associated with reduced quality of life, physical capacity and cardiac function. Continuous positive airway pressure improved respiratory events, sleep quality, left ventricular function and exercise performance. Adaptive servo‐ventilation reduced central respiratory events but was associated with increased mortality in patients with reduced left ventricular function. Nocturnal oxygen therapy improved some respiratory parameters but did not affect quality of life. Structured exercise programs showed benefits for functional status and quality of life.

**Conclusion:**

Obstructive sleep apnea negatively affects the quality of life in people with chronic heart failure. Continuous positive airway pressure is beneficial in selected patients, while adaptive servo‐ventilation should be used cautiously. Personalised approaches are needed.

**Implications for the Profession and/or Patient Care:**

Systematic screening and individualised care plans, including nurse‐led management, are key to improving quality of life and outcomes in this population.

**Impact:**

What problem did the study address? The insufficient understanding of how obstructive sleep apnea influences quality of life in chronic heart failure.

What were the main findings? Some interventions improved quality of life, while others showed safety concerns.

Where and on whom will the research have an impact? The findings guide clinical care for adults with chronic heart failure and coexisting sleep‐disordered breathing.

**Reporting Method:**

We have adhered to the EQUATOR guidelines according to PRISMA.

**Patient or Public Contribution:**

No patient or public contribution.

## 1 Introduction

Chronic heart failure (CHF) is a complex and progressive cardiovascular disease in which the heart is unable to pump blood effectively to meet the body's demands (Roger 2021). It affects millions of people worldwide and continues to impose a significant healthcare burden due to its high prevalence, morbidity and mortality (Vollset et al. 2024). The global prevalence of CHF is estimated to range from 2.1% in the general adult population to 10% in individuals over 70 years (Roger 2021; Vollset et al. 2024). In Europe, CHF affects approximately 2% of the population, while in Asia, it is reported at varying rates from 1.1% to 2.8% (Bozkurt et al. 2023; Feng et al. 2024). In Italy, CHF poses a significant public health challenge, with an estimated prevalence between 1% and 2% in the adult population and over 10% in individuals aged 70 and older. It is one of the leading causes of hospitalisation among elderly patients (Marangoni et al. 2012). Despite advances in medical treatments and improved survival rates, CHF remains a major cause of hospitalisation, with rehospitalization rates as high as 50% (Tung et al. 2016). This condition is associated with numerous symptoms, including dyspnea, fatigue, fluid retention and impaired exercise tolerance, all of which significantly affect the quality of life (QoL) of those affected individuals (Arrigo et al. 2020). One of the most concerning comorbidities in patients with CHF is sleep‐disordered breathing (SDB), particularly obstructive sleep apnea (OSA) and Cheyne‐Stokes respiration (CSR) (Bitter et al. 2011). Sleep apnea is highly prevalent in this population, with studies indicating that between 40% and 70% of individuals with CHF experience some form of sleep apnea (Li et al. 2015). In Europe and Asia, the prevalence of sleep apnea among CHF patients is similarly high, with obstructive sleep apnea being the most common type (Abbasi et al. 2021). This comorbidity not only exacerbates cardiovascular symptoms but also negatively influences quality of life, contributing to increased fatigue, decreased sleepiness and impaired cognitive function (Abbasi et al. 2021; Arrigo et al. 2020; Bitter et al. 2011). Sleep apnea causes intermittent hypoxia during sleep, leading to additional cardiovascular stress and worsening CHF symptoms. This contributes to poorer prognoses, increased hospitalizations, and higher mortality rates (Polecka et al. 2023). The coexistence of CHF and sleep apnea is increasingly recognised as a critical factor influencing patient outcomes (Arrigo et al. 2020; Polecka et al. 2023). Although both conditions are well documented separately, the combined impact of CHF and sleep apnea on QoL has been underexplored (Khayat et al. 2013). Existing studies have primarily focused on the individual cardiovascular and respiratory consequences of each condition, without fully addressing how they jointly affect patients' physical, mental and emotional well‐being (Borkowski and Borkowska 2024; Polecka et al. 2023). Furthermore, much of the literature emphasises physiological outcomes, such as hospitalizations, mortality and clinical parameters (DiCaro et al. 2024; Polecka et al. 2023), while overlooking the broader, multidimensional impact on QoL.

Although some studies have focused on the type of treatment used in CHF patients with OSA (Abbasi et al. 2021; Bitter et al. 2011; Polecka et al. 2023), no systematic review has specifically examined the QoL of this patient population. There is a lack of comprehensive research that integrates both the physiological burden of these comorbidities and patient‐reported outcomes related to QoL. While sleep apnea can exacerbate CHF symptoms, the full extent of its impact on daily functioning, mental health and life satisfaction has not been adequately explored (Jean‐Louis et al. 2008). As a result, healthcare providers lack clear guidance on how to manage the psychological and functional challenges experienced by CHF patients with concomitant sleep apnea.

This systematic review aims to address this knowledge gap by evaluating the impact of OSA on the QoL of patients with CHF, a population frequently affected by sleep‐disordered breathing, which significantly impairs functional status and overall well‐being in patients (Han et al. 2021). In recent years, the promotion of self‐care has become a fundamental component in the management of chronic conditions, with nurses playing a central role in supporting patients' adherence to therapeutic plans and lifestyle changes. Furthermore, caregiver involvement in self‐care has been shown to significantly enhance QoL in CHF patients. This approach, grounded in chronic care models widely applied to other conditions such as diabetes, highlights the need for integrated strategies in the management of CHF complicated by OSA (Caggianelli et al. 2024). Therefore, this review seeks to systematically examine the existing literature, providing a comprehensive analysis of the interaction between OSA and CHF, and identifying clinical and care strategies that may improve prognosis, QoL, and overall well‐being in this vulnerable patient population.

### 1.1 Aims and Research Questions

The aim of this review is to describe the QoL of patients with CHF and coexisting OSA. The research question guiding this review is: How is QoL affected in patients with CHF who also develop OSA?

## 2 Methods

### 2.1 Study Design

This systematic review was conducted in accordance with the updated guidelines of the Preferred Reporting Items for Systematic Reviews and Meta‐Analyses (PRISMA) statement (Moher et al. 2009a, 2009b). To enhance the scientific rigor of the study, the PRISMA checklist (see Data S2) was used, and the research protocol was registered in the PROSPERO database (International Prospective Register of Systematic Reviews) (ID = CRD42025634352).

### 2.2 Eligibility Criteria

The eligibility criteria for this systematic review were defined and justified in alignment with the review's scope and methodology. These criteria were developed through collaborative consensus among the research team, using the PEO model (Population, Exposure, Outcome) as a guiding framework (Davies 2011). The target population consists of patients with CHF. The exposure of interest is the onset of OSA and the measured outcome is the QoL. The review included primary studies such as observational, cohort, cross‐sectional, quasi‐experimental, before‐and‐after studies, randomised controlled trials (RCTs), non‐randomised controlled trials and other intervention studies. Eligible studies focused on patients aged 18 years or older, regardless of gender. Only studies that directly addressed the research question related to sleep apnea in the context of CHF were included. Exclusion criteria were established to maintain the focus and methodological quality of the review. Secondary studies, such as literature reviews, were excluded, as the review aimed to include only primary empirical research. Furthermore, studies that did not follow the IMRAD structure (Introduction—Method—Results—and—Discussion) (Sollaci and Gomes Pereira 2004), such as letters to the editor, commentaries and editorials, were not considered, as these do not provide the necessary methodological rigour for the review analysis. Studies that provided only an abstract without access to the full‐text were also excluded, as they did not offer sufficient data for a detailed evaluation. Furthermore, studies focusing on paediatric or neonatal populations were excluded, as the review targeted adult cardiac patients. Research examining sleep apnea in individuals without cardiovascular disease was also excluded, as it did not align with the central research question. Lastly, studies published in languages other than English or Italian were excluded due to translation limitations.

### 2.3 Information Sources

A thorough literature search was systematically conducted in the following databases to identify relevant records: PubMed (via MEDLINE), CINAHL (via EBSCO), Scopus and Web of Science (via EBSCO).

### 2.4 Search Strategy

As part of the search strategy, the complete set of search terms and Boolean combinations used across all databases is provided in Data S1. Following PRISMA methods (Moher et al. 2009a, 2009b) the study selection process involved two main stages: initial screening of titles and abstracts, followed by full‐text assessment. All potentially relevant studies identified during the search phase were imported into Zotero software to ensure a transparent and traceable process. Duplicate records were removed through a combination of automated Zotero tools (Takats et al. 2025) and manual inspection, addressing potential discrepancies, such as variations in letter casing, that might not have been flagged by the software. After duplicates were excluded, the remaining records were uploaded to Rayyan (Ouzzani et al. 2016). The screening process was conducted by a dedicated research team. A researcher (FG), who was not involved in the selection process, uploaded the records to Rayyan, while two independent reviewers (DD and FL) assessed the studies. This method ensured impartiality, as the reviewers had no prior knowledge of the records. Disagreements between the reviewers were resolved through discussion; if consensus could not be reached, a third reviewer (GM) intervened to resolve the conflict. In the initial phase, titles and abstracts of potentially relevant studies were selected based on inclusion and exclusion criteria. Subsequently, full‐text versions of the selected studies were obtained using various methods, most of which were retrieved through Zotero's functions. The remaining full texts were sourced through online searches or by accessing the journals in which the studies were published. Despite these efforts, five full texts could not be obtained, even after attempts to contact the authors and access the journals directly. Finally, the available full‐text articles were assessed for eligibility according to the predefined criteria.

### 2.5 Data Collection Process

A data extraction framework was employed to systematically gather relevant information from the selected studies, ensuring alignment with the objectives of the systematic review. This framework was developed and adapted based on the most recent edition of the Cochrane Handbook for Systematic Reviews of Interventions (Cumpston et al. 2022). The extraction process was coordinated by a lead researcher (FG) to ensure consistency and consensus in the collected data, thereby promoting accuracy throughout the review. Data were extracted using Microsoft Excel and Zotero software. According to established protocols, two independent reviewers (D.D. and F.L.) extracted data from each study, improving the reliability of the process. Any inconsistencies that emerged during data collection were addressed through a structured discussion involving a third reviewer to resolve discrepancies and ensure alignment.

### 2.6 Data Items

Data collected from selected studies was methodically organised into specific categories for thorough reporting and in‐depth analysis, following the guidelines outlined by the Cochrane methodology (Moher et al. 2009a, 2009b). These categories included: bibliographic information (e.g., authors, publication year), study design, country of origin, research setting, sample size, the condition under investigation (i.e., sleep apnea in patients with chronic heart failure), and the assessment tools used. Additionally, data on QoL outcomes and their respective measurement instruments, the nature of the association (positive, neutral, or negative) and the main findings (e.g., statistical significance of the observed relationships) were systematically recorded.

### 2.7 Evaluation of the Risk of Bias Assessment

According to the PRISMA guidelines (Moher et al. 2009a, 2009b), a comprehensive risk of bias assessment was conducted for each included study. This evaluation was independently performed by two reviewers (D.D. and F.L.) to ensure methodological accuracy and consistency. The most recent versions of the Joanna Briggs Institute (JBI) Critical Appraisal Checklists were used to assess the methodological quality of the included studies (Aromataris et al. 2015). Any disagreements between reviewers were thoroughly discussed with a third researcher (GM) until consensus was reached. This structured process ensured a rigorous and transparent evaluation of study quality and internal validity.

### 2.8 Effect Measures

In accordance with PRISMA guidelines (Moher et al. 2009a, 2009b), the results were synthesised and presented based on quantitative data extracted from the included studies. These data encompassed various statistical indicators, such as means (M), standard deviations (SD), odds ratios (OR), effect size measures (e.g., *r*‐values), correlation coefficients (e.g., Pearson's *r*) and significance levels (*p*‐values), which were used to assess the relationships between the independent variable (patients with OSA) and the outcome (QoL). To preserve the integrity of the original studies, reported significance levels were retained as published, rather than standardised across studies. This approach ensured a consistent and faithful summary of findings in alignment with the objectives of the systematic review.

### 2.9 Synthesis Methods

Despite acknowledging the potential benefits of conducting a meta‐analysis, the wide variation in research methodologies and measurement tools employed across the studies included in this review prevented a combined quantitative analysis, as outlined in the Cochrane Handbook for Systematic Reviews of Interventions (Cumpston et al. 2022). In particular, there was significant heterogeneity in the tools used to measure key variables (such as different instruments that assess QoL) and in the statistical approaches applied to analyze these relationships. These differences resulted in a lack of consistency both methodologically and statistically. As a result, a narrative synthesis was conducted following the guidelines of Synthesis Without Meta‐analysis (SWiM) (Campbell et al. 2020). This approach enabled a transparent and rigorous integration of the data, in line with PRISMA standards (Moher et al. 2009a, 2009b). To explore the complex relationships between the variables, a vote count strategy was employed as recommended by the SWiM guidelines. The level of evidence was evaluated using the Oxford Centre for Evidence‐Based Medicine (OCEBM) grading system. The classification criteria are as follows: 1 = Systematic review of RCTs or a single RCT; 2 = Systematic review of cohort studies, individual cohort studies, or low‐quality RCTs; 3 = Systematic review of case–control studies or individual case–control studies; 4 = Case series or low‐quality cohort and case–control studies; 5 = Expert opinion.

## 3 Results

### 3.1 Study Selection

**FIGURE 1:**
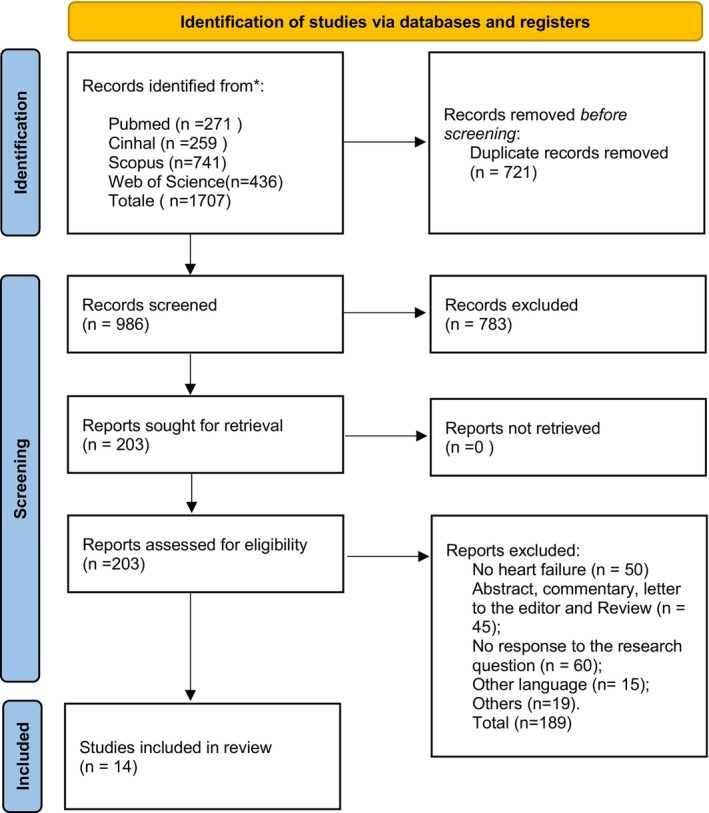
Flowchart studies included.

A total of 1707 records were identified through searches of the biomedical databases PubMed, CINAHL, Scopus and Web of Science. After removing 721 duplicates, 986 records remained and were screened by title and abstract. Of these, 783 records were excluded based on title/abstract screening. Subsequently, 203 full‐text articles were retrieved and assessed for eligibility. Ultimately, 14 studies focusing on the QoL in individuals with CHF and coexisting OSA were included in the review (Figure 1).

### 3.2 Characteristics of the Study

The studies include two cohort studies (Carmona‐Bernal et al. 2008; Lang‐Stöberl et al. 2024), one case–control study (Awotidebe et al. 2017), four cross‐sectional studies (Chen et al. 2009; Mills et al. 2009; Patidar et al. 2011; Redeker et al. 2010) and seven RCTs (Bordier et al. 2015; Cowie et al. 2015; McMillan et al. 2014; Olseng et al. 2017; Philippe 2005; Servantes et al. 2012; Zhao et al. 2017). They were published between 2005 and 2024 and enrolled a total of 2048 participants diagnosed with CHF and various types of SDB, including OSA, central sleep apnea (CSA) and CSR. The sample sizes ranged from 25 to 1325 patients, and most studies included between 30 and 120 participants. In three studies, the total number of patients with preserved left ventricular ejection fraction (LVEF) was 98, whereas the remaining 11 studies included patients with LVEF ≤ 45%. Eleven studies evaluated the effects of specific interventions on SDB and cardiac or QoL outcomes. Commonly used diagnostic tools included complete polysomnography, cardiorespiratory polygraphy and nocturnal oxygen therapy. The apnea‐hypopnea index (AHI) was the primary diagnostic criterion for SDB in all studies, with thresholds for moderate to severe SDB typically defined as AHI ≥ 15 events/h. Therapeutic interventions relied predominantly on ASV (Bordier et al. 2015; Patidar et al. 2011; Philippe 2005), continuous positive airway pressure (CPAP) (McMillan et al. 2014; Philippe 2005; Zhao et al. 2017), nocturnal oxygen therapy (NOT) (Bordier et al. 2015), and structured exercise studies (Servantes et al. 2012). CPAP studies focused primarily on patients with OSA or CSA and demonstrated treatment durations ranging from a few weeks to 6 months (Philippe 2005). ASV studies targeted CSR and CSA‐CSR and included longer follow‐up periods, with the largest study, SERVE‐HF (*n* = 1325), following participants for up to 24 months (Cowie et al. 2015). QoL was assessed using the Minnesota Living with Heart Failure Questionnaire (MLHFQ), the Short Form‐36 (SF‐36), and the Epworth Sleepiness Scale (ESS). Physical function and exercise tolerance were measured by the 6‐min walking test (6MWT), maximum oxygen uptake (VO_2_ max) and wrist actigraphy (Servantes et al. 2012). Cardiac function was monitored using echocardiography‐derived LVEF and New York Heart Association (NYHA) class (Awotidebe et al. 2017), while sleep quality was assessed using polysomnographic indices (AHI, arousals, sleep efficiency) and the Pittsburgh Sleep Quality Index (PSQI) (Redeker et al. 2010). Study populations were generally middle‐aged and older adults (mean age: 58–72 years), with a predominance of male participants (Table 1).

**TABLE 1:** Summary of included studies.

Author(s) and years	Country	Aims/purpose	Population	Methodology	Outcomes	Key findings	Levels of evidence
Carmona‐Bernal et al. (2008)	Spain	To assess the impact of CSR‐CSA on QOL in patients with CHF	90	Cohort study	Five patients (obstructive sleep apnea) were excluded. Of the 85 remaining patients, 25 presented CSR‐CSA. The mean MLHFQ score was higher in patients with CHF and CSR‐CSA (25.8 ± 2.97 vs. 16.6 ± 2.05; *p* = 0.01), and showed a significant yet moderate correlation with the AHI. A lower mean FOSQ score was obtained for the group of patients with CHF and CSR CSA (78.4 ± 4.31 vs. 88.47 ± 2.4; *p* = 0.03), showing a weak negative correlation with the AHI	Congestive heart failure; Central sleep apnea; Minnesota living with heart failure questionnaire; Functional outcomes of sleep questionnaire	Level 3
Patidar et al. (2011)	India	The present study was undertaken to assess the prevalence of OSA and associated QOL among CHF patients and to ascertain the relationship of OSA with excessive daytime sleepiness and selected demographic, clinical, and anthropometric characteristics	Fifty CHF patients and 50 healthy controls	Cross selectional	Excessive daytime sleepiness was significantly associated with OSA in CHF patients (*p* = 0.02). Clinical severity (New York Heart Association class) and duration of illness were not significantly associated with OSA. Increased body mass index and neck circumference were the significant risk factors responsible for OSA. Quality of life of CHF patients was poor, and OSA had a significantly negative impact on the already compromised QOL in CHF patients as well as in individuals with no CHF	Body mass index, congestive heart failure, excessive daytime sleepiness, obstructive sleep apnea, quality of life	Level 3
Bordier et al. (2015)	Berlin	This paper studies the short‐ and long‐term effects of NOT on sleep apnea in CHF	51	RCT	No significant difference was observed between baseline and 6 months in the no NOT group. In the NOT group, AHI decreased from 36.8 ± 2.6 events/h at baseline to 20.8 ± 3.0 at 24 h and to 18.3 ± 2.4 at 6 months (both P)	Heart failure. Sleep apnea. Hypoxia. Oxygen therapy	Level 2
Servantes et al. (2012)	Brasil	To evaluate the effects of home‐based exercise for patients with chronic heart failure and sleep apnoea and to compare two different training programmes	50	RCT	Results: Of the 50 patients enrolled in the study, 45 completed the programme. Clinical events: Group 1 (one death), Group 2 (one myocardial infarction), Group 3 (one death and two strokes). None were training related. Training groups showed improvement in all outcomes evaluated and the adherence was an important factor (Group 1 = 98.5% and Group 2 = 100.2%, *p* ¼ 0.743). Untrained Group 3 demonstrated significant decrease or no change on measurements after 3 months without training. Conclusion: Home‐based exercise training is an important therapeutic strategy in chronic heart failure patients with sleep apnoea, and strength training resulted in a higher increase in muscle strength and endurance	Group 1 (one death), Group 2 (one myocardial infarction), Group 3 (one death and two strokes). None were	Level 2
Philippe (2005)	France	To compare compliance with and effectiveness of ASV versus CPAP in patients with CSA with CSR and with congestive heart failure in terms of the AHI, QoL, and LVEF over 5 months	25	RCT	Both ASV and CPAP decreased the AHI but, noticeably, only ASV completely corrected CSA‐CSR, with AHI below 10/h. At 3 months, compliance was comparable between ASV and CPAP; however, at 6 months compliance with CPAP was significantly less than with ASV. At 6 months, the improvement in quality of life was higher with ASV and only ASV induced a significant increase in LVEF	ASV was superior to CPAP in correcting CSA‐CSR, improving LVEF and enhancing quality of life; better long‐term compliance	Level 2
Cowie et al. (2015)	England	We investigated adaptive servo‐ventilation's effects in patients with heart failure with reduced ejection fraction and predominantly central sleep apnea	1325	RCT	In the adaptive servo‐ventilation group, the mean AHI at 12 months was 6.6 events per hour. The incidence of the primary endpoint did not differ significantly between the adaptive servo‐ventilation group and the control group (54.1% and 50.8%, respectively; hazard ratio, 1.13; 95% confidence interval [CI], 0.97 to 1.31; *p* = 0.10). All‐cause mortality and cardiovascular mortality were significantly higher in the adaptive servo‐ventilation group than in the control group (hazard ratio for death from any cause, 1.28; 95% CI, 1.06 to 1.55; *p* = 0.01; and hazard ratio for cardiovascular death, 1.34; 95% CI, 1.09 to 1.65; *p* = 0.006)	ASV reduced AHI but increased all‐cause and cardiovascular mortality in patients with HFrEF and central sleep apnea. ASV not recommended for this population	Level 2
Mills et al. (2009)	USA	This study characterised sleep in HF and determined associations with QoL	74	Cross selectional	Findings provide evidence that in addition to functional status and ongoing fatigue, poorer quality of life in HF is independently related to the severity of sleep‐disordered breathing	OSA in heart failure patients is associated with poorer health‐related quality of life and more depressive symptoms; screening and treatment for OSA may benefit this population	Level 3
Chen et al. (2009)	Taiwan	The objective of this study was to investigate predictors of self‐reported sleep disturbances in Taiwanese people with heart failure	125	Cross selectional	Self‐reported sleep disturbances were prevalent (74%) among people with heart failure in Taiwan. Five predictors were identified using hierarchical multiple regression analyses with forward methods, accounting for 26.9% of variance in sleep disturbances. They were education, New York Heart Association functional classification, perceived health, HRQOL social functioning and physical symptoms. After controlling for demographics, heart failure characteristics and health‐related characteristics, the analysis showed that two variables of HRQOL accounted for 9.8% of the variance in sleep disturbances	Health‐related quality of life & heart failure & sleep disturbance	Level 3
Zhao et al. (2017)	Boston	The long‐term effect of CPAP on HRQOL in patients with high cardiovascular disease risk and OSA without severe sleepiness is uncertain	169	RCT	CPAP improved several domains of HRQOL including bodily pain (treatment effect 9.7 [95% confidence interval, CI 3.9 to 15.4]; *p* = 0.001), vitality (5.7 [95% CI 1.5 to 9.9]; *p* = 0.008), general health (8.2 [95% CI 3.7 to 12.7]; *p* < 0.001), physical functioning (5.5 [95% CI 1.1 to 10.0]; *p* = 0.016), and the physical health summary score (3.3 [95% CI 1.4 to 5.3]; *p* = 0.001). CPAP also resulted in less daytime sleepiness (mean change in ESS −1.0 point [95% CI −2.0 to −0.0]; *p* = 0.040)	Sleep apnea, CPAP, quality of life, clinical trial, sleepiness	Level 2
Redeker et al. (2010)		To evaluate characteristics of SDB; clinical and demographic correlates of SDB; and the extent to which SDB explains functional performance and symptoms in stable heart failure patients receiving care in structured HF disease management programs	170	Cross selectional	There were no statistically significant relationships between SDB and daytime symptoms or self‐reported sleep, despite poorer objective sleep quality in patients with SDB	Heart failure; sleep disordered breathing; sleep apnea, actigraphy; fatigue, depression, sleep	Level 3
Awotidebe et al. (2017)	Nigeria	This study investigated the relationship between FC and SpQ of patients with CHF and apparently HCs	100	Case control	Patients had a significantly lower FC and poorer SpQ than HCs, 4.6 0.5 versus 11.3 1.6 mL/kg/min (*t* Z 3.452; *p* Z 0.001) and 8.74 1.6 versus 3.8 1.3 (*t* Z 5.371; *p* Z 0.001), respectively. HCs were about five times more likely to walk longer distance [odds ratio (OR), 4.8; confidence interval (CI), 2.0–11.1] and had a better heart rate (OR, 2.8; CI, 1.4–5.3) than patients. SpQ had a significant negative correlation with FC of patients (*r* Z 0.362; *p* Z 0.001) but a significant positive correlation with HCs (*r* Z 0.481; *p* Z 0.041). Furthermore, there were significant correlations between FC and body mass index in both groups (CHF: *r* Z 0.247, *p* Z 0.022; HCs: *r* Z 0.321, *p* Z 0.040)	Chronic heart failure; Functional capacity; Healthy control; Sleep quality	Level 3
Olseng et al. (2017)	Oslo, Norway	The aim of this study was to investigate if quality of life improved in chronic heart failure patients with Cheyne ‐Stokes respiration treated with adaptive servo—ventilation in nurse ‐led heart failure clinic.	102	RCT	Adaptive servo‐ventilation improved quality of life‐scores both in a per protocol analysis and in an intention to treat analysis. QoL measured with MLHFQ. AHI, mask adherence, monthly nurse follow‐up	Chronic heart failure, quality of life, Cheyne‐Stokes respiration, nurse‐led heart failure clinic, adaptive servo‐ventilation	Level 2
McMillan et al. (2014)	London	The therapeutic and economic benefits of CPAP for moderate to severe OSA syndrome have been established in middle‐aged people; however, the benefits in older people are unknown. This trial was designed to address this evidence gap	278	RCT	231 (83%) completed the trial. 140 patients were allocated to and received CPAP plus BSC and 138 were allocated to and received BSC only. CPAP reduced ESS by 2.1 points (95% CI –3.0 to −1.3; *p* < 0.0001) at 3 months for 124 (89%) of 140 patients compared with 124 (90%) of 138 patients given BSC, and by 2.0 points (−2.8 to −1.2; *p* < 0.0001) at 12 months for 116 patients compared with 122 patients given BSC. The eff ect was greater in patients with higher CPAP usage or higher baseline ESS. CPAP improved objective sleepiness (*p* = 0.024), mobility (*p* = 0.029), total cholesterol (*p* = 0.048) and LDL cholesterol (*p* = 0.042) at 3 months, but these were not sustained at 12 months	CPAP significantly reduced subjective sleepiness at 3 and 12 months (−2.1 and −2.0 ESS points). Improved quality of life (SF‐36 vitality). Positive effects on mobility, cholesterol and LDL at 3 months but not maintained at 12. No effect on blood pressure, CV events, cognitive function or mood. Low mean CPAP use (1.5–2.5 h/night). CPAP marginally cost‐effective (QALY +0.01 with EQ‐5D). CPAP also recommended for older adults with OSA, especially with high ESS	Level 2
Lang‐Stöberl et al. (2024)	Austria	To investigate the impact of SDB on hemodynamic regulation, HRV, BRS and survival in patients with chronic heart failure	58	Cohort study	HRV measurements, BRS, hemodynamic parameters, long‐term survival, sleep quality, AHI, O2 saturation, PSQI, ESS, MLHFQ	SDB prevalent in 63.8%; patients with severe SDB showed lower BRS and higher LF/HF ratio; worse survival in patients with moderate–severe SDB (age‐dependent); no relevant impact of CPAP therapy on survival	Level 3

*Note:* The Level of evidence was evaluated using the Oxford Centre for Evidence‐Based Medicine (OCEBM) grading system (Howick et al. 2023). The classification criteria are as follows: 1 = Systematic review of Randomised Controlled Trials (RCTs) or a single RCT; 2 = Systematic review of cohort studies, individual cohort studies, or low‐quality RCTs; 3 = Systematic review of case–control studies or individual case–control studies; 4 = Case series or low‐quality cohort and case–control studies; 5 = Expert opinion.

Abbreviations: AHI, apnoea–hypopnoea index; ASV, adaptive servo‐ventilation; BRS, baroreceptor reflex sensitivity; CHF, chronic heart failure; CPAP, continuous positive airway pressure; CSA, central sleep apnoea syndrome; CSR, Cheyne‐Stokes respiration; HCs, healthy controls; HF, heart failure; HF, heart failure; HRQOL, health‐related quality of life; HRV, heart rate variability; LVEF, left ventricular ejection fraction; NOT, nocturnal oxygen therapy; OSA, obstructive sleep apnoea; OSA, sleep apnea; QoL, Quality of Life; RCT, Randomised Controlled Trial; SDB, disturbed sleep; SDB, sleep‐disordered breathing.

### 3.3 Quality Assessment and Level of Evidence

The quality of the included studies was assessed using the most recent version of the Joanna Briggs Institute (JBI) Critical Appraisal Checklists. Methodological quality varied, ranging from moderate to high. Of the 14 included studies, seven were classified as high quality, while seven were rated as moderate. The highest‐rated studies were those by Redeker et al. (2010), Servantes et al. (2012), Zhao et al. (2017), McMillan et al. (2014), Mills et al. (2009), Cowie et al. (2015) and Lang‐Stöberl et al. (2024) that met between 85% and 100% of the quality criteria. All studies demonstrated methodological transparency and data integrity, which supported their inclusion in the synthesis. A summary of the risk of bias assessment and quality scores is provided in Table 2. According to the OCEBM levels of evidence (Howick et al. 2023), seven studies were classified as level 2 and seven as level 3.

**TABLE 2:** Quality appraisal of studies included.

Authors and years	Q1	Q2	Q3	Q4	Q5	Q6	Q7	Q8	Q9	Q10	Q11	Q12	Q13	Result (*n*, %)	Level of quality
**Checklist for case control studies critical appraisal tools for use in JBI systematic reviews**															
Awotidebe et al. (2017)	Y	Y	N	Y	Y	N	N	Y	Y	Y				(7; 70%)	MODERATE
**Checklist for cohort study critical appraisal tools for use in JBI systematic reviews**															
Lang‐Stöberl et al. (2024)	Y	Y	Y	Y	Y	Y	Y	Y	N	N	Y			(9; 81%)	HIGH
Carmona‐Bernal et al. (2008)	N	Y	Y	Y	Y	N	Y	Y	N	Y	Y			(8; 75%)	MODERATE
**Checklist for randomised controlled trial critical appraisal tools for use in JBI systematic reviews**															
Cowie et al. (2015)	Y	Y	Y	N	N	Y	Y	Y	Y	Y	Y	Y	Y	(11; 85%)	HIGH
McMillan et al. (2014)	Y	Y	Y	N	N	Y	Y	Y	Y	Y	Y	Y	Y	(11; 85%)	HIGH
Olseng et al. (2017)	Y	Y	Y	N	N	Y	N	Y	Y	Y	Y	Y	Y	(10; 76%)	MODERATE
Philippe (2005)	Y	Y	Y	N	N	Y	Y	Y	U	Y	Y	Y	Y	(10; 76%)	MODERATE
Zhao et al. (2017)	Y	Y	Y	N	U	Y	Y	Y	Y	Y	Y	Y	Y	(11; 85%)	HIGH
Bordier et al. (2015)	Y	Y	Y	U	U	Y	U	Y	Y	Y	Y	Y	Y	(10; 76%)	MODERATE
Servantes et al. (2012)	Y	Y	Y	Y	N	Y	N	Y	Y	Y	Y	Y	Y	(11; 85%)	HIGH
**Checklist for cross sectional studies critical appraisal tools for use in JBI systematic reviews**															
Chen et al. (2009)	Y	Y	Y	Y	N	N	Y	Y						(6; 75%)	MODERATE
Redeker et al. (2010)	Y	Y	Y	Y	Y	Y	Y	Y						(8; 100%)	HIGH
Mills et al. (2009)	Y	Y	Y	Y	Y	Y	N	Y						(7; 87%)	HIGH
Patidar et al. (2011)	Y	Y	Y	Y	Y	N	N	Y						(6; 75%)	MODERATE

*Note:* Checklist for case series and case report in critical appraisal tools. Level of quality (Y); Low < 50%; Moderate 50% = > and < 70%; High > = 70%.

Abbreviations: JBI, Joanna Briggs Institute; N, NO; N/A, Not Applicable; U, Unclair; Y, Yes.

### 3.4 Synthesis of Findings

Across the reviewed studies, SDB was consistently associated with negative impacts on QoL, functional capacity and cardiac function in patients with CHF. SDB, which includes OSA, CSA and CSR, is highly prevalent in this population, with reported rates ranging from 50% to over 80% depending on the phenotype and diagnostic method (Lang‐Stöberl et al. 2024; Redeker et al. 2010). In five RCTs, CPAP has been shown to significantly reduce the apnea‐hypopnea index (AHI) and improve subjective and objective results (Bordier et al. 2015; Carmona‐Bernal et al. 2008; Chen et al. 2009; Patidar et al. 2011). In an RCT involving 41 patients with CSA, CPAP reduced AHI from baseline and improved Epworth Sleepiness Scale (ESS) scores and QoL measures (*p* < 0.01) (Chen et al. 2009). Similarly, in OSA populations, CPAP improved sleep efficiency, reduced arousal index and enhanced functional performance (6MWT distance and self‐reported physical function) (Awotidebe et al. 2017; Lang‐Stöberl et al. 2024). In some cohorts, CPAP also led to significant improvements in LVEF, particularly in patients with moderate to severe CHF (Zhao et al. 2017). ASV was more effective than CPAP in reducing central respiratory events. In a prospective study of 25 patients with CSA‐CSR, ASV reduced AHI to < 10 events/h and increased LVEF by an average of 5% over 6 months (Bordier et al. 2015). ASV also showed better adherence rates and higher QoL scores compared to CPAP at follow‐up. However, data from a large multicenter RCT (*n* = 1325) indicated that in patients with reduced LVEF (≤ 45%), ASV, despite reducing AHI to 6.6 events/h after 12 months, was associated with increased all‐cause mortality (HR = 1.28; *p* = 0.01) and cardiovascular mortality (HR = 1.34; *p* = 0.006), raising concerns about its safety in this subgroup (Cowie et al. 2015). In a single‐center RCT involving 51 patients (NYHA class II–III, LVEF ≤ 45%) with sleep apnea (AHI ≥ 15 events/h), 19 patients were randomised to nocturnal oxygen therapy (NOT; 3.0 L/min) and 14 to no treatment. Follow‐up polygraphy showed that in the NOT group, AHI decreased from 36.8 ± 2.6 at baseline to 20.8 ± 3.0 at 24 h and 18.3 ± 2.4 at 6 months (both *p* < 0.0001), primarily due to reductions in central AHI (from 23.3 ± 2.8 to 6.1 ± 1.4, *p* < 0.0001). The oxygen desaturation index decreased from 33.0 ± 5.2 to 9.3 ± 2.6 events/h (*p* < 0.0001). However, NOT had no significant effect on obstructive/mixed AHI, QoL, LVEF, or NYHA class over 6 months (Bordier et al. 2015). Two interventional studies (*n* = 62 and *n* = 36) evaluated structured exercise programs, with or without CPAP, and reported significant improvements in physical capacity (measured by peak VO_2_ and 6MWT), QoL scores, and reductions in fatigue and sleep disturbance severity (Awotidebe et al. 2017; Servantes et al. 2012). Functional and symptom‐related correlates of SDB were also explored. A cross‐sectional study of 170 stable CHF patients found that severe SDB was independently associated with a fourfold increased risk of poor physical function (OR = 4.15; 95% CI: 1.19–14.57), and CSA was linked to significantly reduced daytime mobility (OR = 4.09; 95% CI: 1.23–13.62), independent of age, sex and LVEF (Redeker et al. 2010). Another study of 58 CHF patients found that an AHI > 30 events/h was associated with reduced baroreceptor reflex sensitivity and an elevated low‐to‐high frequency ratio in heart rate variability (*p* < 0.05), suggesting autonomic imbalance and reduced survival. However, age was identified as a confounding factor in this association (Lang‐Stöberl et al. 2024).

## 4 Discussion

This systematic review confirms that the coexistence of CHF and SDB contributes to a multidimensional deterioration in patients' QoL, with significant repercussions on physical, functional, psychological and social dimensions (Menon and Kalra 2024). The presence of SDB exacerbates the burden of CHF not only by worsening respiratory function, but also by intensifying symptoms such as fatigue, dyspnea, excessive daytime sleepiness and sleep disturbances. Collectively, these clinical features impair patients' perceived well‐being and autonomy, reflecting a profound decline across multiple QoL domains (Lang‐Stöberl et al. 2024). Clinically, CPAP has shown promise in improving several aspects of QoL, particularly in patients with OSA and significant daytime sleepiness (Bordier et al. 2015; Carmona‐Bernal et al. 2008; Chen et al. 2009; Patidar et al. 2011; Zhao et al. 2017). However, the use of ASV must be approached with caution in patients with HFrEF, in light of the increased mortality risk observed in the SERVE‐HF trial (Cowie et al. 2015). Non‐ventilatory interventions, such as structured exercise programs and NOT, have shown selective benefits in enhancing functional capacity and improving respiratory parameters, further underscoring the need for personalised therapeutic strategies (Bordier et al. 2015; Servantes et al. 2012). A critical aspect that emerged from the results is the central role of nursing interventions in the integrated management of patients with CHF and SDB. Nursing follow‐up clinics related to the management of these pathologies have demonstrated a significant impact in improving therapeutic adherence, optimising home management of ventilator devices and supporting patients' self‐care behaviours (Awotidebe et al. 2017; Olseng et al. 2017; Vellone et al. 2020), showing favourable effects on functional capacity, QoL and the consequent improvement of nocturnal respiratory parameters (Servantes et al. 2012). Nursing strategies, including patient education, motivational coaching, and follow‐up via telemonitoring, are contained among the principles of the Chronic Care Model (Francesconi et al. 2019; Limonti et al. 2025; Riegel et al. 2022) already widely applied in the management of chronic diseases such as diabetes and CHF (Caggianelli et al. 2024). The development of advanced nursing competencies is essential for assessing respiratory symptoms, managing CPAP and ASV devices, promoting self‐care and addressing lifestyle‐related risk factors such as obesity and physical inactivity. These skills are crucial for improving both clinical outcomes and patient‐reported QoL (Awotidebe et al. 2017; Caggianelli et al. 2024; Lazzeroni and Riccò 2023; Olseng et al. 2017). In addition, the findings highlight the need for public health strategies aimed at promoting early screening for SDB in patients with CHF and supporting the implementation of home‐based care programs. This can be facilitated by the involvement of family and community nurses, thereby integrating SDB management into comprehensive, multidisciplinary care pathways for CHF patients (Scrimaglia et al. 2024). Promoting a multidimensional, patient‐centered model of care, guided by specialised nursing interventions, emerges as a strategic priority to reduce hospital readmissions and improve the overall well‐being of this vulnerable patient population.

### 4.1 Future Research, Policy and Practice

Future research should focus on phenotype‐specific trials to assess the long‐term safety and efficacy of CPAP and ASV in CHF patients with SDB (Menon and Kalra 2024). Given that ASV may be harmful in individuals with reduced LVEF, there is a clear need for personalised treatment strategies. Clinical guidelines should promote routine screening for SDB in patients with CHF to enable timely diagnosis and intervention [47,48]. Adjunct therapies, such as structured exercise programs, have shown additional benefits in improving both QoL and physical capacity, and should be further explored in combination with ventilatory treatments. The role of NOT remains uncertain and requires additional investigation to determine its effectiveness and appropriate patient populations. Moreover, future studies should examine the impact of autonomic dysfunction in this population and its association with SDB severity, as it may serve as a prognostic marker and inform the development of more targeted interventions (Hadaya and Ardell 2020).

### 4.2 Limitation

This review has several limitations that should be considered when interpreting the results. Although the JBI was used, the methodological quality of the included studies is moderate. Significant heterogeneity in study design, sample size, outcome measures and duration of follow‐up limited the strength and generalizability of the conclusions. The overall level of evidence, assessed according to OCEMB criteria, showed level 2 studies, as controlled studies, equaling the number of other level 3 studies. Additionally, potential publication and language bias cannot be excluded, as only peer‐reviewed articles in English were included. Most studies were conducted in Western countries and in highly specialised clinical settings, which may limit the applicability of the results to other healthcare contexts, particularly in low‐resource environments or in non‐specialist care settings. Furthermore, while some interventions demonstrated short‐term improvements in respiratory parameters and QoL, their long‐term effectiveness remains unclear due to the limited follow‐up duration in the majority of studies.

## 5 Conclusions

This systematic review highlights the impact of OSA on QoL in patients with CHF, an area still underrepresented in clinical research and marked by significant methodological heterogeneity. Available evidence supports the use of CPAP, particularly in patients with symptomatic OSA, as an effective strategy to improve multiple dimensions of QoL. However, the use of ASV in patients with HFrEF and CSA requires careful risk–benefit evaluation due to its potential association with increased mortality. Psychosocial factors such as mood disorders, reduced autonomy and social isolation also emerge as important contributors to QoL deterioration in this population. In this context, personalised exercise programs and structured, nurse‐led follow‐up interventions appear to be crucial in supporting overall well‐being and enhancing patient engagement. Nurses play a central role in assessing QoL, educating patients and caregivers, promoting adherence to ventilatory therapies and integrating behavioural and lifestyle interventions. The complexity of managing patients affected by both CHF and OSA calls for advanced nursing competencies, particularly in chronic disease management, home‐based care planning and motivational health communication. For this reason, training programs and health policies should aim to reinforce the role of nurses as coordinators of personalised, multidimensional care models capable of meeting the needs of a fragile and multimorbid population.

## Author Contributions

**Francesco Limonti:** conceptualization, writing – original draft; **Giovanni Cangelosi:** writing – review and editing; **Diaratou Dabre:** conceptualization, writing – original draft; **Gianluca Marino:** writing – review and editing; **Arcangelo Correra:** writing – review and editing; **Sara Morales Palomares:** review and editing; **Nicola Ramacciati:** supervision; **Francesco Gravante:** conceptualization, writing – review and editing.

## Funding

The authors have nothing to report.

## Conflicts of Interest

The authors declare no conflicts of interest.

## Supporting information


**Data S1:** nop270385‐sup‐0001‐supinfo01.docx.

**Data S2:** nop270385‐sup‐0002‐supinfo2.docx.

## Data Availability

The data files collected are available on request from the corresponding author.
