# Supplementary material for: Quality of Life in Chronic Heart Failure With Obstructive Sleep Apnea: A Systematic Review
Source: Nurs Open. 2025 Nov 28;12(12):e70385. doi: 10.1002/nop2.70385 (PMC12662253; doi:10.1002/nop2.70385)
Supplement: Supplementary file 1 — Data S1: nop270385‐sup‐0001‐supinfo01.docx. [file NOP2-12-e70385-s001.docx]

| **Search** | **Query** | **Results** |
| --- | --- | --- |
| #13 | **(((('Heart Failure') OR ('Cardiac Failure')) OR ('Heart Failure patient')) AND (('Sleep Apnea') OR ('Apnea, Sleep'))) AND ((('Quality of Life') OR ('Health-Related Quality Of Life')) OR ('HRQOL'))** | [271](https://pubmed.ncbi.nlm.nih.gov/?term=%28%28%28%28%27Heart+Failure%27%29+OR+%28%27Cardiac+Failure%27%29%29+OR+%28%27Heart+Failure+patient%27%29%29+AND+%28%28%27Sleep+Apnea%27%29+OR+%28%27Apnea%2C+Sleep%27%29%29%29+AND+%28%28%28%27Quality+of+Life%27%29+OR+%28%27Health-Related+Quality+Of+Life%27%29%29+OR+%28%27HRQOL%27%29%29&sort=) |
| #12 | **(('Quality of Life') OR ('Health-Related Quality Of Life')) OR ('HRQOL')** | [580,218](https://pubmed.ncbi.nlm.nih.gov/?term=%28%28%27Quality+of+Life%27%29+OR+%28%27Health-Related+Quality+Of+Life%27%29%29+OR+%28%27HRQOL%27%29&sort=) |
| #11 | **'HRQOL'** | [580,218](https://pubmed.ncbi.nlm.nih.gov/?term=%27HRQOL%27&sort=) |
| #10 | **'Health-Related Quality Of Life'** | [580,073](https://pubmed.ncbi.nlm.nih.gov/?term=%27Health-Related+Quality+Of+Life%27&sort=) |
| #9 | **'Quality of Life'** | [580,073](https://pubmed.ncbi.nlm.nih.gov/?term=%27Quality+of+Life%27&sort=) |
| #8 | **((('Heart Failure') OR ('Cardiac Failure')) OR ('Heart Failure patient')) AND (('Sleep Apnea') OR ('Apnea, Sleep'))** | [3,372](https://pubmed.ncbi.nlm.nih.gov/?term=%28%28%28%27Heart+Failure%27%29+OR+%28%27Cardiac+Failure%27%29%29+OR+%28%27Heart+Failure+patient%27%29%29+AND+%28%28%27Sleep+Apnea%27%29+OR+%28%27Apnea%2C+Sleep%27%29%29&sort=) |
| #7 | **('Sleep Apnea') OR ('Apnea, Sleep')** | [60,661](https://pubmed.ncbi.nlm.nih.gov/?term=%28%27Sleep+Apnea%27%29+OR+%28%27Apnea%2C+Sleep%27%29&sort=) |
| #6 | **'Apnea, Sleep'** | [58,658](https://pubmed.ncbi.nlm.nih.gov/?term=%27Apnea%2C+Sleep%27&sort=) |
| #5 | **'Sleep Apnea'** | [60,661](https://pubmed.ncbi.nlm.nih.gov/?term=%27Sleep+Apnea%27&sort=) |
| #4 | **(('Heart Failure') OR ('Cardiac Failure')) OR ('Heart Failure patient')** | [352,459](https://pubmed.ncbi.nlm.nih.gov/?term=%28%28%27Heart+Failure%27%29+OR+%28%27Cardiac+Failure%27%29%29+OR+%28%27Heart+Failure+patient%27%29&sort=) |
| #3 | **'Heart Failure patient'** | [211,080](https://pubmed.ncbi.nlm.nih.gov/?term=%27Heart+Failure+patient%27&sort=) |
| #2 | **'Cardiac Failure'** | [352,459](https://pubmed.ncbi.nlm.nih.gov/?term=%27Cardiac+Failure%27&sort=) |
| #1 | **'Heart Failure'** | [327,555](https://pubmed.ncbi.nlm.nih.gov/?term=%27Heart+Failure%27&sort=) |

**Supplementary Table A. History of the development of the PubMed search string, detailing the sequential formulation, refinement, and expansion of the search terms used throughout the review’s search strategy design.**

**Supplementary Table B. Final search strings used across all databases included in the review: PubMed, Scopus, Web of Science, and CINAHL.**

| **Database** | **Query** | **Results** |
| --- | --- | --- |
| PUBMED | (((('Heart Failure') OR ('Cardiac Failure')) OR ('Heart Failure patient')) AND (('Sleep Apnea') OR ('Apnea, Sleep'))) AND ((('Quality of Life') OR ('Health-Related Quality Of Life')) OR ('HRQOL')) | 271 |
| SCOPUS | ('Heart Failure') AND (('Sleep Apnea') OR ('Apnea, Sleep') AND ((('Quality of Life') | 741 |
| WEB OF SCIENCE | ('Heart Failure') AND ('Sleep Apnea') OR ('Apnea, Sleep') AND ('Quality of Life') | 436 |
| CINAHL | ('Heart Failure') AND ('Sleep Apnea') OR ('Apnea, Sleep') AND ('Quality of Life') | 259 |
| **TOTAL** |  | 1707 |
